# Enhancing Recruitment Using Teleconference and Commitment Contract (ERUTECC): study protocol for a randomised, stepped-wedge cluster trial within the EFFECTS trial

**DOI:** 10.1186/s13063-017-2367-8

**Published:** 2018-01-08

**Authors:** Erik Lundström, Eva Isaksson, Per Wester, Ann-Charlotte Laska, Per Näsman

**Affiliations:** 1 Karolinska Institutet, Department of Clinical Neuroscience, Neurology, Karolinska University Hospital, Solna, 171 76 Stockholm, Sweden; 2 Karolinska Institutet, Department of Clinical Neuroscience, Neurology, Karolinska University Hospital, Building R3:04, Solna, 171 76 Stockholm, Sweden; 3 Department of Clinical Sciences, Danderyd Hospital, Karolinska Institutet, 182 88 Stockholm, Sweden; 4 Department of Public Health and Clinical Medicine, Umeå University, 901 87 Umeå, Sweden; 5 Department of Clinical Sciences, Danderyd Hospital, Karolinska Institutet, 18288 Stockholm, Sweden; 6 Centre for Safety Research, KTH Royal Institute of Technology, 100 44, Stockholm, Sweden

**Keywords:** Stroke, Randomised controlled trial, RCT, Recruitment, Randomised stepped-wedge cluster trial

## Abstract

**Background:**

Many randomised controlled trials (RCTs) fail to meet their recruitment goals in time. Trialists are advised to include study recruitment strategies within their trials.

EFFECTS is a Swedish, academic-led RCT of fluoxetine for stroke recovery. The trial’s primary objective is to investigate whether 20 mg fluoxetine daily compared with placebo for 6 months after an acute stroke improves the patient’s functional outcome. The first patient was included on 20 October 2014 and, as of 31 August 2017, EFFECTS has included 810 of planned 1500 individuals. EFFECTS currently has 32 active centres.

The primary objective of the ERUTECC (Enhancing Recruitment Using Teleconference and Commitment Contract) study is to investigate whether a structured teleconference re-visit with the study personnel at the centres, accompanied by a commitment contract, can enhance recruitment by 20% at 60 days post intervention, compared with 60 days pre-intervention, in an ongoing RCT.

**Methods:**

ERUTECC is a randomised, stepped-wedge cluster trial embedded in EFFECTS. The plan is to start ERUTECC with a running-in period of September 2017. The first intervention is due in October 2017, and the study will continue for 12 months. We are planning to intervene at all active centres in EFFECTS, except the five top recruiting centres (*n* = 27). The rationale for not intervening at the top recruiting centres is that we believe they have reached their full potential and the intervention would be too weak for them. The hypothesis of this study is that a structured teleconference re-visit with the study personnel at the centres, accompanied by a commitment contract, can enhance recruitment by 20% 60 days post intervention, compared to 60 days pre-intervention, in an ongoing RCT.

**Discussion:**

EFFECTS is a large, pragmatic RCT of stroke in Sweden. Results from the embedded ERUTECC study could probably be generalised to high-income Western countries, and is relevant to trial management and could improve trial management in the future. It might also be useful in clinical settings outside the field of stroke.

**Trial registrations:**

The ERUTECC study was registered in the Northern Ireland Hub for Trials Methodology Research Studies Within a Trial repository (SWAT58) on 30 April 2017.

ClinicalTrials.gov, ID: NCT02683213. Retrospectively registered on 2 February 2016.

**Electronic supplementary material:**

The online version of this article (doi:10.1186/s13063-017-2367-8) contains supplementary material, which is available to authorized users.

## Background

### Introduction

Many randomised controlled trials (RCTs) fail to meet their recruitment goals in time [1, 2]. A study of 114 multicentre trials in the UK showed that 45% failed to reach 80% of their recruitment goal. Less than one third of the trials recruited their original target number of participants within the time originally specified, and around one third had to be extended in terms of time and resources [3], and it has been identified as the highest priority to find methods to enhance recruitment to the RCT [4]. There are a few trials that have sought to evaluate different strategies for recruiting patients. However, these studies are small, and some are hypothetical, making the interpretation unclear [5]. Qualitative interventions within trials to improve recruitment have been developed in the UK [6], and in addition, online resources [7, 8] are available. In spite of that, we need to know more about the barrier and success factors for trials in recruiting patients and especially in RCTs. Given these facts, trialists are advised to include study recruitment strategies within their trials [5].

One alternative is to embed trials of recruitment interventions within host trials. An embedded recruitment trial is defined as [9]:

‘…a RCT in which an intervention (or several interventions) to enhance recruitment outcomes are tested in the context of another RCT (or several RCTs) known as the host RCT(s)’.

The EFFECTS (Efficacy oF Fluoxetine – a randomisEd Controlled Trial in Stroke, Clinical Trials number NCT02683213) study seeks to investigate whether 20 mg fluoxetine daily compared with placebo for 6 months after acute stroke improves patients’ functional outcome [10]. The EFFECTS study is a multicentre trial aiming to recruit 1500 patients in Sweden. The study collaborates with two other investigator-led studies, FOCUS (UK) and AFFINITY (Australia/New Zealand/Vietnam). Each trial is funded independently and intends to report its own results [10].

The first patient in EFFECTS was included on 20 October 2014. Our primary recruitment goal was that each centre should randomise at least two patients per month. In reality, there are huge differences between centres regarding recruitment. Looking back between June 2016 and May 2017, for instance, only three centres have achieved the recruitment goal: Danderyd Hospital, Karolinska University Hospital Solna and Sundsvall Hospital, while another three (Mora General Hospital, Alingsås General Hospital and Skaraborg Hospital Skövde) came close. In fact, these six centres have so far included half of the individuals. This pattern – that a few centres have included the majority of individuals – has been consistent since the start, irrespective of different strategies. The recruitment rate per month and centre is updated in real time and is available in the public domain [11].

### Rationales for the study

Currently, the EFFECTS trial includes 30 patients per month [11], and recruitment projections would take the trial beyond its current funding. Thus, a new strategy is needed.

After talking to the principal investigators (PIs) at the centres, and to trialists in the UK with similar studies, we are confident that five of the top recruiters (Danderyd Hospital, Karolinska University Hospital Solna, Sundsvall Hospital, Mora General Hospital and Skaraborg Hospital Skövde) have reached their full potential, and little would be gained from the planned intervention.

We have asked the centres via a survey, and have also discussed this in person at investigator meetings, what they consider to be the most important barrier to recruitment for trials. They have said that the most important barrier is the lack of time for the physician responsible to identify the right patients and to carry out the study-specific procedures at baseline. The second most important factor is the lack of time for all other study personnel. They have a high clinical workload at their clinic and do not have any time specifically dedicated to working with clinical trials. It is the head of department (verksamhetschef) who is responsible for finances and personnel. Without the support of the head of department there will hardly be any change in recruitment. We will, therefore, invite the head of department (verksamhetschef) at the local centre to the teleconference in addition to the PI and the research nurse. Hopefully, we can work together to identify what could be done to provide more opportunities at the clinic to do research for both physicians and nurses within working hours. We have chosen a teleconference because it is time effective and less expensive than a face-to-face meeting. In addition, we hypothesised that a commitment contract would make it more personal for the local centre.

We have followed the 2013 SPIRIT (Standard Protocol Items: Recommendations for Interventional Trials) Checklist [12] in conjugation with the 2013 SPIRIT explanation and elaboration guidance for protocols of clinical trials [13] (Additional file 1).

### Objectives of the study

#### Primary objective

The primary objective of this study is to investigate whether a structured teleconference re-visit with the study personnel at the centres, accompanied by a commitment contract, can enhance recruitment by 20% during 60 days post intervention, compared with 60 days pre-intervention, in an ongoing RCT.

#### Secondary objectives

- Recruitment rate 61–120 days post teleconference
- We will compare the recruitment 60 days post teleconference with a baseline inclusion from 1 Sep 2017 to 31 Oct 2017 for all centres. The purpose is to see if there is any bias or impact during the course of the study

The secondary objective must be seen as exploratory due to low power.

### Hypothesis

Does a structured teleconference re-visit with the study personnel (PI, at least one research nurse, and the head of department) at the centres, accompanied by a commitment contract, enhance recruitment by 20% during 60 days post intervention, compared with 60 days pre-intervention in the EFFECTS study?

### The rationale for the trial design

We have chosen the stepped-wedge cluster randomised study design [14] for three reasons. First, it is not possible for us to carry out the intervention at 14–15 centres at the same time. Second, we believe that all medium and low recruiting centres could gain from the intervention, and in a stepped-wedge cluster design all centres are exposed. Every step provides before and after observations, and every step switches from control to become exposed to the intervention. Third, we have noticed a seasonal variation in recruitment. During the Christmas and Easter holidays, and especially during the summer, recruitment is falling. This intervention gives a realistic view of recruitment throughout the whole year. To the best of our knowledge, only one randomised stepped-wedge study in the field of stroke has been done [15].

## Methods

### Study settings

EFFECTS has been running for almost 3 years, and we know the recruitment rate per centre and month. Initially we had 35 centres. Three centres – Karolinska Hospital Huddinge, Visby General Hospital and Högsbo Rehabilitation Hospital – have been closed due to low recruitment mainly because of a lack of PI. Of the 32 centres, we have identified five centres that have achieved our goal, that is recruiting two or more patients per month. They are the top recruiters: Danderyd Hospital, Karolinska University Hospital Solna, Skaraborg Hospital Skövde, Mora General Hospital and Sundsvall Hospital. We will not intervene at these centres because we believe that they have reached their full potential and the intervention is too weak for them. Not including the top recruiters, this leaves us with 27 centres in this study.

**Fig. 1:**
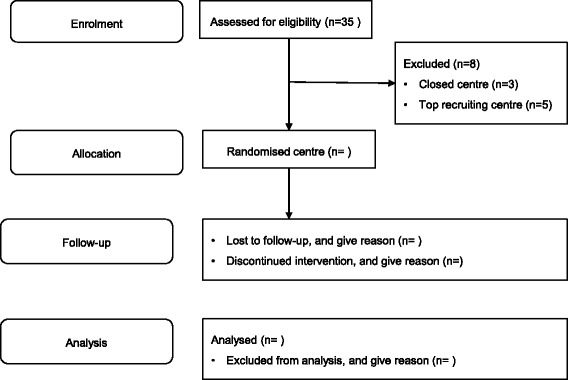
Consolidated Standards of Reporting Trials (CONSORT) flow diagram for the ERUTECC study. ERUTECC is a randomised, stepped-wedged trial within the EFFECTS study. EFFECTS has initiated 35 centres, of which 32 are active. We will exclude the five top recruiting centres because we believe they have reached their full potential, and the planned intervention is too weak

Figure 1 shows a Consolidated Standards of Reporting Trials (CONSORT) flow diagram [16] of the study. Hence, ERUTECC is a randomised, stepped-wedge cluster design [14] study. We made some minor changes to the CONSORT flow diagram layout.

### Study type

We will use a randomised, stepped-wedge cluster design, where every step provides data before and after intervention, but not at the same point in time.

**Fig. 2:**
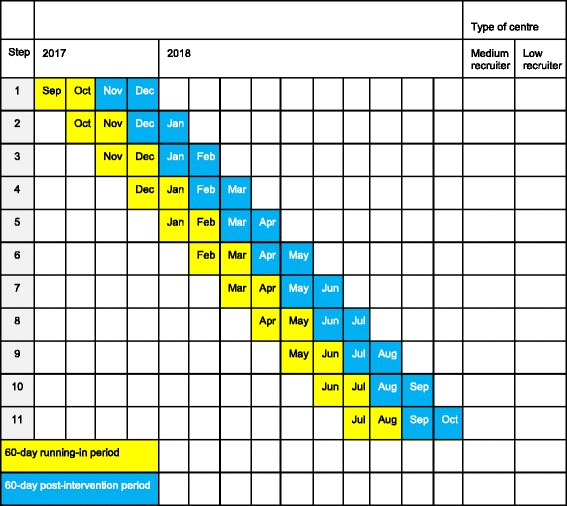
The randomised, stepped-wedge cluster design for the ERUTECC study. In ERUTECC, we use a stepped-wedge cluster design. First the centre is classified as low or medium recruiting. Second, the centres are randomised in each class ensuring that at least one low and one medium recruiting centre is included in every step. Each centre has a 60-day running-in period (yellow), followed by a 60-day post-intervention period (blue). The intervention (teleconference) is done after the 60-day running-in period, and every step provides data before and after intervention, but not at the same point in time. We will add up all patients for all 11 steps in the 60-day running-in period and compare this with the inclusion rate for all centres’ 60-day post randomisation period

**Fig. 3:**
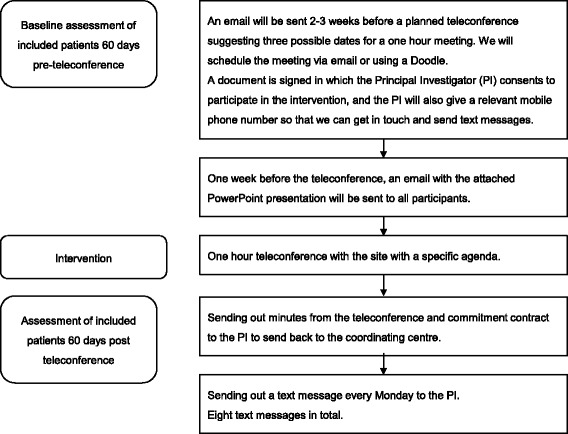
The flow of participants in the ERUTECC study. In ERUTECC, each centre follows the same flow, with a 60-day period of running in, and a 60-day post-intervention period (teleconference). The figure shows one step. We will add up all patients for all 11 steps in the 60-day running-in period and compare this with the inclusion rate for all centres’ 60-day post randomisation period

Figure 2 illustrates the stepped-wedge cluster study in our study. As the name indicates, the intervention has 11 different steps, and the schema takes the form of a wedge. Figure 3 is a schematic diagram of the time schedule of enrolment, interventions (including any run-ins and washouts) and assessments for the participating centre.

In ERUTECC, all centres have a 60-day running-in period and a 60-day post-intervention period. Our plan is to start in September 2017 and finish in October 2018.

The 27 sites will be divided into two categories: medium and low recruiters. The rationale for this is that we do not want to risk all medium recruiters falling into the same step, for example the summer period, which usually a low-recruiting period. The centre will be randomised in terms of the order in which intervention will be performed, leading to (at least) one medium and (at least) one low recruiter in each of the 11 steps (Fig. 2).

All centres in the EFFECTS study, their classification, type and numbers of patients are listed in Table 1.

**Table 1:** Centres in EFFECTS as of 31 August 2017

Centre	Type of centre	Number of stroke patients per year^a^	First included patient in EFFECTS (yyyy-mm-dd)	Total number of patients recruited	Percentage of number of patients recruited^b^
01 Danderyd Hospital	SU at hosp	837	11/11/2014	110	14%
02 Karolinska University Hospital Solna	SU at univ hosp	542	10/20/2014	98	12%
03 Skaraborg Hospital Skövde	SU at hosp	422	10/20/2014	56	7%
04 Hässleholm Hospital	SU at hosp	203	3/23/2015	32	4%
05 Uppsala University Hospital	SU at univ hosp	496	4/20/2015	39	5%
06 Karolinska University Hospital Huddinge	SU at univ hosp	488	4/8/2015	15	2%
07 Mora General Hospital	SU at hosp	226	4/15/2015	49	6%
08 Falu General Hospital	SU at hosp	510	5/13/2015	12	1%
09 Lidköping	SU at hosp	162	10/6/2015	12	1%
10 Capio St Göran’s	SU at hosp	697	6/24/2015	51	6%
11 Visby General Hospital	SU at hosp	132	11/4/2015	7	1%
12 Norrland University Hospital	SU at univ hosp	355	9/22/2015	12	1%
13 Kristianstad Central Hospital	SU at hosp	334	9/24/2015	11	1%
14 Norrtälje Hospital	SU at hosp	165	12/9/2015	1	0%
15 Helsingborg General Hospital	SU at hosp	391	11/18/2015	15	2%
16 Skåne University Hospital Malmö	SU at univ hosp	478	12/18/2015	16	2%
17 Halland Hospital Halmstad	SU at hosp	374	12/1/2015	36	4%
18 Mälar Hospital Eskilstuna	SU at hosp	246	2015-22-23	10	1%
19 Rehab Station Stockholm	Neuro RH	^c^	11/24/2015	4	0%
20 Skåne University Hospital Lund	SU at univ hosp	634	2/29/2016	9	1%
21 Sundsvall Hospital	SU at hosp	486	12/18/2015	66	8%
22 Sahlgrenska University Hospital	SU at univ hosp	789	4/15/2015	16	2%
23 Högsbo Rehabilitation Hospital	Neuro RH	^c^	3/4/2016	2	0%
24 Stora Sköndal Neurological Rehabilitation Clinic	Neuro RH	^c^	1/22/2016	13	2%
25 Östersund Hospital	SU at hosp	339	3/10/2016	21	3%
26 Alingsås General Hospital	SU at hosp	223	2/25/2016	34	4%
27 Ängelholm Hospital	SU at hosp	232	3/15/2016	16	2%
28 Stockholm Nursing Home	Neuro RH	^c^	4/4/2016	13	2%
29 Örebro Rehabilitation Clinic	Neuro RUH	^c^	10/10/2016	10	1%
30 Norra Älvsborg County Hospital Trollhättan	SU at hosp	699	12/3/2016	4	0%
31 Bromma Geriatric Clinic	Ger RH	^c^	11/24/2016	1	0%
32 Västmanland Hospital Västerås	SU at hosp	411	1/18/2017	9	1%
33 Dalen Hospital	Ger RH	^c^	5/22/2017	2	0%
34 Lindesberg General Hospital	SU at hosp	117	6/15/2017	2	0%
35 Hudiksvall Hospital	SU at hosp	187	4/12/2017	6	1%
Total				810	100%

The centres are numbered in the order they were initiated. Closed centre denotes that the centre is closed for EFFECTS and not included for ERUTECC. *Abbreviations: SU at hosp* Stroke unit at hospital, *SU at univ hosp* Stroke unit at university hospital, *Neuro RH* Neurological rehabilitation hospital, *Neuro RHU* Neurological university hospital, *Ger RH* Geriatric rehabilitation hospital

^a^Numbers of patients in the stroke unit according to the National Swedish Quality Register [19]

^b^Number of patients included divided by total number of stroke patients during active participating time the EFFECTS study as of 31 August 2017

^c^Rehabilitation hospitals/units. The exact number of stroke is not part of the Riksstroke statistics

### Inclusion criteria

1. Active centre in the EFFECTS study. We will invite all the study personnel at each centre. Study personnel are defined as all persons listed at the delegation list at the time for the invitation. For the meeting to happen, at least the PI, one research nurse and the head of department at the centre/hospital/unit must attend the meeting (*n* = 3).

### Exclusion criteria

1. Top recruiting centre in the EFFECTS study, i.e. Danderyd Hospital, Karolinska University Hospital Solna, Sundsvall Hospital, Mora General Hospital or Skaraborg Hospital Skövde.

### Preparation before the intervention

This is what we will do before the intervention (teleconference):

1. One person, i.e. the trial manager, trial manager assistant or a PhD student, will contact the local PI or the research nurse approximately 2–3 weeks before a planned meeting and suggest three possible dates for a 1-h meeting. We will schedule the meeting via email or using a Doodle
2. A document is signed in which the PI consents to participate in this intervention study. In this document, the PI also gives a relevant mobile phone number that we can use to get in touch and send text messages
3. One week before the meeting, an email with the agenda and PowerPoint presentation attached will be sent to all participants
4. One day before, a text message and an email will be sent to all participants as a reminder of the meeting

### The intervention – a structured teleconference

This is a teleconference between the chief investigator (CI) plus the trial manager and the study personnel. The meeting requires that at least the PI, one research nurse and the head of department (verksamhetschef) must attend the meeting. If they are not all able to attend, the meeting will be rescheduled. Ideally, as many members as possible listed on the delegation list will attend the meeting.

The agenda

(a) Opening of the meeting (1 min,). Chair: trial manager. Secretary: chief investigator. Keeping track of the time: chief investigator
(b) Presentation of all at the meeting (2 min, all)
(c) Presentation of EFFECTS, PowerPoint (4 min, chief investigator)
The rationale of the study (one slide)
An update of overall recruitment (one slide)
What we need to do – our aim (one slide)
An update of recruitment at the local site (one slide)
Discussion of local barriers (5–7 min, all)
What can we do at our local centre? (5–7 min, all)
  Discussion with the head of department about barriers and what can be done (5 min, all)
  Where do we go from here? Formulate a commitment contract, minimum one item, maximum three items (5–7 min, trial manager)
  Closing and summary of the meeting (2 min, trial manager)

### Post meeting agenda

1. Sending out the summary of the meeting and the Commitment Contract to the PI to sign and send back to the Trial Office
2. Sending out a text message every Monday to the PI and research nurse during the whole period as a friendly reminder

### Refusing to participate in the study

If a centre refuses to participate in the study, the reason for this is noted. We will include the centre as ‘intention-to-treat’.

### Statistics

First, we excluded the top recruiting centres. Second, the remaining centres were categorised as low or medium recruiters. Third, in each category (low and medium) the order of the intervention was randomised using a computer-generated allocation sequence using SAS version 9:4 by our statistician, PN. Neither the intervention nor the order of the intervention is communicated in advance. The intervention in known by the Steering Committee, ACL (author of this manuscript) and the Regional Ethical Committee, and is not communicated to the centres. The order of the allocation is known by three persons (PN, EI and EL), and is kept in an Excel file behind a secure firewall at Stockholm County Council. The centre will be contacted by the trial manager (EI) 3–5 weeks before the planned intervention. For obvious reasons, the intervention is not blinded.

### Sample size and randomisation

The study has been running for almost 3 years and we know the recruitment rate per centre and month. Among the 32 centres in EFFECTS, we have identified five centres that have achieved our goal, i.e. recruiting two or more patients per month. These are the top recruiters (Danderyd, Karolinska Solna, Skövde, Sundsvall and Mora). We will not intervene with these centres because we believe that they have reached their full potential and the intervention is too weak for them. Not including the top recruiters leaves us with 27 centres in this study.

### Blinding

The participants in the EFFECTS study are not aware of the embedded study. We will not mention the intervention or in what order we will do the intervention outside the group responsible for ERUTECC. For obvious reasons, there is no blinding of the intervention. The centres will not be informed that we are measuring numbers of randomised patients before and after the teleconference, but they are fully aware that we want to enhance recruitment. The exact numbers of recruitments per centre has been available in the public domain through a link that has been updated in real time since the start of the EFFECTS study.

### Statistical methods

We will compare the numbers of included individuals 60 days before the teleconference with the numbers of subjects 60 days post intervention. The null hypothesis is that there will be no difference before and after. We consider a 20% increase in recruitment rate as clinical relevant, although it is arbitrarily chosen.

Statistical example:

- Sixty days pre-teleconference: 60 individuals
- Sixty days post-teleconference: 78 individuals
- That is, 78/60 = 1.3; a 30% increase in recruitment

In EFFECTS, we have a screening list in which we note all eligible individuals who have been considered as candidates for EFFECTS. If these patients are not part of the study, the reason is noted. Usually the reasons are administrative and non-willingness. We will compare the numbers of randomised patients with the eligible patients on the screening list.

The outcome of the study is the number of included patients per centre. Inclusion is available through our electronic randomisation system, and we will keep a separate log for every teleconference. If a centre refuses to be part of ERUTECC, we will still include the centre in the analysis (intention-to-treat). In addition, we will also do a ‘per-protocol analysis’.

### Planned subgroup analyses:

- Are there any differences in recruitment rate between a medium recruiting centre versus a low recruiting centre?
- To what extent does the size of the stroke unit contribute to the number and percentage of patients included? Are there any differences between large stroke units versus small stroke units?
- Are there any differences between stroke units versus rehabilitation centres?
- Are there any differences between university hospitals versus non-university hospitals?
- Are there any differences between experienced centres versus non-experienced centres?

### Recruitment

We aim to start this study in September 2017. At that time, we will have 27 active centres, not including the five top recruiters.

### Explanation of the randomised, stepped-wedge design

- Step 1 starts with a 60-day running-in period beginning in September 2017, and consists of at least one medium and one low recruiting centre. The teleconference is performed at the end of October 2017, and the observation follows 60 days post intervention
- Step 2 starts with a 60-day running-in period beginning in October 2017, and consists of at least one medium and one low recruiting centre. The teleconference is performed at the end of November 2017, and the observation follows 60 days post intervention

### Harm

It is hard to anticipate any harm from ERUTECC. The host trial, EFFECTS, has specific monitoring performed by the Karolinska Trial Alliance. The monitor is independent and the Karolinska Trial Alliance follows a specific monitor plan, which, for example, checks every patient consent. The participants in ERUTECC will received no extra benefits for being part of the study and there will be no compensation for those who claim that they have suffered harm from trial participation.

### Problems

Anticipated problems.

- It is possible that the intervention will develop during the course of the study. At the beginning we will have a clear agenda, but it is possible that we will learn through the process, and that the intervention will become a little different
- It is also possible that the participants at the centres that have not yet been intervened with will find out about the interventions and will change their pattern of behaviour before the intervention takes place
- The organisation of the intervention will be challenging because it involves more than 27 centres over a period of 1 year
- At some centres, the head of the department will not be able to join during the structured telephone conference
- It is possible some centres will refuse to be a part of the intervention and this will interfere with the results

## Discussion and generalisability

EFFECTS is a pragmatic study of stroke and the largest RCT study of stroke in Sweden. The result from this embedded study could probably be generalised to high-income countries, like Sweden. The result could be useful in clinical settings outside the field of stroke, and if our study is positive, the results could be applicable to a wide range of RCT studies. The study will add knowledge about the management of RCT studies and recruitment.

### Limitations

- One limitation is that we will not include the top recruiters. However, we believe that the high recruiting centres have reached their maximum level of recruitment and the intervention will only have a minor effect
- It is possible that the intervention is too weak to have positive results. After all, we are trying to change the pattern of behaviour for over 60 persons and behaviour change is one of the hardest things to accomplish
- Since we started the EFFECTS study, we have already tried different things to identify barriers and find ways to enhance recruitment, so it may be that there is nothing more to be done
- We are planning to carry out multiple sub-group analyses. The results must be interpreted with caution

## Trial status

The main study – EFFECTS – included its first patient on 20 October 2014, and as of 31 October 2017, 881 patients have been included. The last patient is estimated to be recruited in March 2019, with the last follow-up 1 year later. The ERUTECC will start in November 2017 (Fig. 2) and continue for 1 year.

## Additional file

Additional file 1: Standard Protocol Items: Recommendation for Interventional Trials (SPIRIT) 2013 Checklist for the ERUTECC study. (PDF 133 kb)

## Abbreviations

CONSORT: Consolidated Standards of Reporting Trials
EFFECTS: Efficacy oF Fluoxetine – a randomisEd Controlled Trial in Stroke
ERUTECC: Enhancing Recruitment Using Teleconference and Commitment Contract
RCT: Randomised controlled trial
SPIRIT: Standard Protocol Items: Recommendations for Interventional Trials
SWAT: Study Within a Trial repository

## References

[1] Vale C, Stewart L, Tierney J (2005). UK Coordinating Committee for Cancer Research National Register of Cancer. Trends in UK cancer trials: results from the UK Coordinating Committee for Cancer Research National Register of Cancer Trials. Br J Cancer.

[2] McDonald AM, Knight RC, Campbell MK, Entwistle VA, Grant AM, Cook JA (2006). What influences recruitment to randomised controlled trials? A review of trials funded by two UK funding agencies. Trials.

[3] Campbell MK, Snowdon C, Francis D, Elbourne D, McDonald AM, Knight R, et al. Recruitment to randomised trials: strategies for trial enrollment and participation study. The STEPS study. Health Technol Assess. 2007;11:iii, ix–105. 10.3310/hta11480 17999843

[4] Tudur Smith C, Hickey H, Clarke M, Blazeby J, Williamson P (2014). The trials methodological research agenda: results from a priority setting exercise. Trials.

[5] Treweek S, Lockhart P, Pitkethly M, Cook JA, Kjeldstrøm M, Johansen M, et al. Methods to improve recruitment to randomised controlled trials: Cochrane systematic review and meta-analysis. BMJ Open. 2013;3. Available from: http://onlinelibrary.wiley.com/doi/10.1002/14651858.MR000013.pub5/abstract;jsessionid=DC197780DFADE29C891FB61BBD7E6199.f03t04. 10.1136/bmjopen-2012-002360 PMC3586125 23396504

[6] Donovan JL, Rooshenas L, Jepson M, Elliott D, Wade J, Avery K (2016). Optimising recruitment and informed consent in randomised controlled trials: the development and implementation of the Quintet Recruitment Intervention (QRI). Trials.

[7] ORRCA: Home [Internet]. Available from: http://www.orrca.org.uk. Accessed 29 Oct 2017.

[8] Trial Forge—A systematic way to improve trial efficiency [Internet]. Trial Forge. Available from: http://www.trialforge.org/. Accessed 29 Oct 2017.

[9] Madurasinghe VW, Sandra Eldridge on behalf of MRC START Group and Gordon Forbes on behalf of the START Expert Consensus Group (2016). Guidelines for reporting embedded recruitment trials. Trials.

[10] Mead G, Hackett ML, Lundström E, Murray V, Hankey GJ, Dennis M (2015). The FOCUS, AFFINITY and EFFECTS trials studying the effect(s) of fluoxetine in patients with a recent stroke: a study protocol for three multicentre randomised controlled trials. Trials.

[11] Effects [Internet]. Available from: https://dcnapp1.dcn.ed.ac.uk/effects/stats/effects_stats.asp. Accessed 6 Sep 2017.

[12] Chan A-W, Tetzlaff JM, Altman DG, Laupacis A, Gøtzsche PC, Krleža-Jerić K (2013). SPIRIT 2013 statement: defining standard protocol items for clinical trials. Ann Intern Med.

[13] Chan A-W, Tetzlaff JM, Gøtzsche PC, Altman DG, Mann H, Berlin JA (2013). SPIRIT 2013 explanation and elaboration: guidance for protocols of clinical trials. BMJ.

[14] Hemming K, Haines TP, Chilton PJ, Girling AJ, Lilford RJ (2015). The stepped wedge cluster randomised trial: rationale, design, analysis, and reporting. BMJ.

[15] Maxwell AE, Dennis M, Rudd A, Weir CJ, Parker RA, Al-Shahi SR (2017). Promoting Recruitment using Information Management Efficiently (PRIME): study protocol for a stepped-wedge cluster randomised controlled trial within the REstart or STop Antithrombotics Randomised Trial (RESTART). Trials.

[16] Consort—The CONSORT Flow Diagram [Internet]. Available from: http://www.consort-statement.org/consort-statement/flow-diagram. Accessed 6 Sept 2017.

[17] ICMJE | Home [Internet]. Available from: http://www.icmje.org. Accessed 17 Sept 2017.

[18] KI ELN—the electronic notebook [Internet]. Available from: http://ki.se/en/staff/ki-eln-the-electronic-notebook. Accessed 17 Sept 2017.

[19] Årsrapport Stroke och TIA 2016, Table 17, Page 58 (in Swedish) [Internet]. Available from: http://www.riksstroke.org/wpcontent/. Accessed 21 Dec 2017.

